# Editorial: Photomedicine

**DOI:** 10.3389/fmed.2019.00161

**Published:** 2019-07-16

**Authors:** Peter Wolf, Frank R. de Grujil

**Affiliations:** ^1^Research Unit for Photodermatology, Department of Dermatology, Medical University of Graz, Graz, Austria; ^2^Department of Dermatology, Leiden University Medical Center, Leiden, Netherlands

**Keywords:** ultraviolet radiation, phototherapy, immune suppression, microbiome, psoriasis, itch, carcinogenesis, photoprotection

Photodermatology is the scientific discipline that deals with how sunlight or parts of it, in particular the ultraviolet (UV) band, affects the skin, our directly visible, frontier organ facing our environment. Although this discipline would appear well within the domain of our every-day experience, many of the basic processes involved are still not fully charted and understood. With regard to therapeutic approaches, the term of photomedicine has been coined, also because some of the effects of light go far beyond the skin and light administration is also used in medicine in general. This special issue aims to present a selection of topics to provide a bird's eye view of the field.

An area of broad public interest is UV protection. Sondenheimer and Krutmann discuss protection of the skin to wavelengths beyond UV by a novel generation of topical agents that boost protective mechanisms of the skin. Parrado et al. consider the possiblity of providing systemic protection by agents taken orally. The protection pertains to sunburn in the short term but to skin cancer in the long term. de Gruijl and Tensen present an overview of how our understanding of the UV pathogenesis of skin carcinoma has grown, in particular the plausible involvement of skin stem cells. And Arisi et al. delve into at times confusing body of data on how solar UV could contribute to raising melanomas, the most aggressive skin cancer.

The skin as a pivotal organ in immunity has become in many ways the essence of photodermatology. Photoimmunology is recognized as a distinguished field of research ever since the discovery of suppressive effects on cellular immunity from UV exposure. In this perspective, recent developments in phototherapy are discussed by Vieyra-Garcia and Wolf. Patra et al. present a novel view on the skin immune system in relation to the skin's microbiome and possible effect thereon from UV exposure; an evidently complicated but promising field of research. As a possibly related issue, Lembo and Raimondo present the advances that have been made in recent years in understanding the pathophysiology of polymorphic light eruption, the most common form of photodermatoses. In particular, a possible central role of an interplay between the immune system, its defense through antimicrobial peptides combined with an inadequate suppression of adaptive immunity against UV responses, on the one hand, and putative “photoantigen(s)” from UV-modified proteins released from (apoptotic) cells, on the other hand.

Besides, many forms and aspects of phototherapy are presented. Ibbotson provides an excellent perspective on the main indications for use of narrowband UVB (311–313 nm) and psoralen and UVA (PUVA) photochemotherapy and provides comparative information on these important dermatological treatments, which despite of the introduction of biologics continue to remain invaluable for many conditions such as psoriasis, atopic eczema, vitiligo, and cutaneous T cell lymphoma. Gambichler and Schmitz focus on the administration and therapeutic mechanisms of ultraviolet A1 (UVA1, 340–400 nm) for fibrosing conditions such as localized scleroderma, lichen sclerosus, systemic sclerosis, nephrogenic systemic fibrosis, and chronic graft-vs.-host-disease (GVHD) of the skin. In contrast, to the other phototherapeutic modalities UVA1 seems to induce changes in fibroblast cytokine production such as transforming growth factor- ß/Smad signaling and interleukin 6, leading to upregulation of collagenase activity, ultimately resulting in less tissue fibrosis. Legat thoroughly discusses the antipruritic effect of phototherapy. Pruritic skin diseases are another area in which phototherapy has remained a mainstay though new drugs, such as the anti-IL31RA antagonist nemolizumab among others, are emerging for itch treatment. It is fascinating to learn that UV may directly affect cutaneous sensory nerve fibers or, through blockage of mediator release (including IL-31) from skin-infiltrating cells, indirectly modulate nerve fiber function as well as the transmission of itch to the central nervous system, inducing the clinically evident antipruritic effect of phototherapy.

Last but not least, Cho et al. give a superb overview on the most complex form of phototherapy, extracorporeal photopheresis (ECP), from a technical point of view. The treatment is a therapeutic gold standard for patients with Sézary syndrome, a systemic form of T cell lymphoma that clinically presents with severe erythroderma. The disease is characterized by abnormal mononuclear cells, which appear in the skin, lymph nodes, and peripheral blood, where those cells and other cells are hit by ECP. Importantly, ECP is also a recommended second-line treatment in steroid-refractory GVHD. The induction of regulatory T cells seems to be the major driver of response in ECP-treated patients.

In sum, the collection of the papers of this special issue of photomedicine illustrates the beauty of the field and teaches how the different phototherapeutic modalities are useful and valuable for the patients, but also how their administration and mechanistic investigation leads to a better understanding of disease mechanisms, allowing ultimately the development of novel and advanced treatment strategies.

## Author Contributions

Both authors listed have made a substantial and direct contribution to the work, and approved it for publication.

### Conflict of Interest Statement

The authors declare that the research was conducted in the absence of any commercial or financial relationships that could be construed as a potential conflict of interest.

